# Occult hepatitis B virus infection in HIV positive patients at a tertiary healthcare unit in eastern India

**DOI:** 10.1371/journal.pone.0179035

**Published:** 2017-06-07

**Authors:** Debraj Saha, Ananya Pal, Neelakshi Sarkar, Dipanwita Das, Jason T. Blackard, Subhasish Kamal Guha, Bibhuti Saha, Runu Chakravarty

**Affiliations:** 1 ICMR Virus Unit, Kolkata, ID & BG Hospital Campus, Kolkata, West Bengal, India; 2 Division of Digestive Diseases, University of Cincinnati College of Medicine, Cincinnati, Ohio, United States of America; 3 Calcutta School of Tropical Medicine, Kolkata, West Bengal, India; Saint Louis University, UNITED STATES

## Abstract

Occult HBV infection (OBI), defined by the presence of HBV DNA in absence of hepatitis B surface antigen (HBsAg), is a significant concern in the HIV-infected population. Of 441 HIV+/HBsAg- patients analyzed, the overall prevalence of OBI was 6.3% (28/441). OBI was identified in 21 anti-HBc positives (17.8%), as well as among those who lacked any HBV-specific serological markers (2.2%). Comparison with HIV/HBV co-infection revealed that the levels of CD4, ALT, and HBV DNA were significantly lower during occult infection. Discrete differences were also observed with respect to quasispecies divergence. Additionally, subgenotype D1 was most frequent in occult infection, while D2 was widespread during chronic infection. The majority (~90%) of occult D1 sequences had the sQ129R mutation in the surface gene. This study highlights several distinct features of OBI in India and underscores the need for additional HBV DNA screening in HIV-positive individuals.

## Introduction

Hepatitis B virus (HBV) infection continues to be a global health concern despite the presence of an effective vaccine. Worldwide, approximately 2 billion people have been infected with HBV at some stage of life, of them ~248 million are chronic carriers of the virus [1]. Chronic or overt HBV infection is characterized by the presence of hepatitis B surface antigen (HBsAg) in serum for at least six months. In contrast, occult HBV infection (OBI) is defined by the presence of HBV DNA in the liver (with detectable or undetectable HBV DNA in the serum) of individuals testing negative for HBsAg by currently available assays [2]. In fact, a true OBI remains HBsAg negative during the entire course and generally possesses very low viral titres [<200 IU/ml] [2, 3]. The presence of antibody to hepatitis B core antigen (anti-HBc) has been used previously as a predictive marker for the detection of OBI [2]; however, OBI also occurs in patients who are negative for all HBV serum markers [4–6].

Co-infection with HBV is frequent among the human immunodeficiency virus (HIV) infected population since they share similar transmission routes [7]. The risk of developing OBI is higher in certain populations, including HIV-positive individuals [8]. Multiple studies have investigated OBI in this high-risk cohort and reported variable prevalence rates between 0%-89.5% [9, 10]. Notably, in countries like South Africa, Iran, Lebanon and UK, a high incidence of OBI had been reported among HIV-positive patients [11–15].

OBI represents a significant diagnostic challenge that has severe clinical consequences. It is associated with transmission *via* hemodialysis, blood donation or organ transplantation [16–20]. Although HIV patients are not ideal candidates for organ or blood donation, there remains a possibility of transmitting HIV and/or HBV either through sexual encounters, needle sharing or other risk behaviors [21]. In fact, the immune-suppressed condition found in HIV infection may even result in reactivation of OBI into overt hepatitis B infection since the regulation of HBV replication by host defenses is compromised [22]. Furthermore, OBI has also been identified as a potential risk factor for the development of HCC [23–25]. In spite of such manifestations, the underlying molecular mechanisms responsible for OBI are not well known. One of the major reasons for the lack of HBsAg may be attributed to mutations found within the surface gene of the HBV genome, which may directly affect HBsAg antigenicity, production, and/or secretion [26–29].

India is intermediately endemic for both HIV and HBV. Our previous study identified a high rate of chronic HBV infection among HIV-infected patients in eastern India [30]. Although a few reports are available on OBI/HIV co-infection from India [31, 32], detailed evaluation of molecular heterogeneity of the occult HBV strains are not well documented. In this context, *Panigrahi et*.*al* had previously studied the partial surface gene of OBI patients with HIV infection from eastern India, though the sample number was only restricted to five [33]. Therefore, lack of any comprehensive study on the mutational profile of HBV or its related quasispecies variability from the OBI/HIV co-infected population makes this an understudied reservoir of HBV replication in India.

## Materials and methods

### Ethics statement

This work was a part of the study approved by “The Institutional Ethical Committee, National Institute of Cholera and Enteric Diseases (ICMR)”. Written informed consent was obtained from all study participants in their native language.

### Study subjects

For the current analysis, 1198 HIV-infected patients, including both antiretroviral (ART)-naive and –experienced subjects, were enrolled from the ART clinic of the Calcutta School of Tropical Medicine (Kolkata) between October 2010 and May 2014. All study participants were part of a research project (BT/ PR14485/Med/29/203/2010) carried out at ICMR Virus Unit, Kolkata. They were screened for HBsAg and different HBV serological markers including HBV DNA. Based on availability of plasma, treatment and HBsAg status, a total of 441 ART-naive HBsAg negative/HIV positive subjects were included.

To compare genomic characteristics of occult versus chronic HBV infection in the HIV population, 69 treatment-naive HIV/HBV co-infected subjects who were positive for HBV DNA (HBsAg positive—Chronic control population) were included. They were also a part of the aforesaid project (BT/ PR14485/Med/29/203/2010) and visited the ART clinic during a similar period. Of these 69 HIV/HBV co-infected samples, 39 had complete HBV surface gene sequencing performed previously [34]. Clinical parameters including CD4 T-cell count, alanine aminotransferase (ALT), and aspartate aminotransferase (AST) values were abstracted at the time of enrolment.

### Serological testing

HBV specific enzyme-linked immunosorbent assay (ELISA) was performed for the detection of HBsAg, anti-HBs (antibody to hepatitis B surface antigen), and anti-HBc (Total) (Bio-Rad, France). All serological assays were performed according to manufacturer’s instruction. The analytical sensitivity for the HBsAg assay was estimated to be 0.025 IU/ml (CI 95%: 0.019–0.037 IU/ml).

### DNA extraction, HBV DNA detection and quantification

After performing the immunoassays for anti-HBc, the HBsAg negative population (441 patients) was stratified into two groups: anti-HBc positive and anti-HBc negative. DNA from each of the anti-HBc reactive samples was extracted using the QIAamp DNA Blood Kit (Qiagen, Hilden, Germany) with 400μl of initial plasma and eluting in 80μl of elution buffer. These samples were then screened for the presence of HBV DNA using a nested polymerase chain reaction (PCR) for two different regions of the HBV genome—surface and basal core promoter/precore region—as described previously (assay lower detection limit: 100copies/ml) [34]. In accordance with an earlier report from South Africa, only samples that could be amplified for both genomic segments were considered as HBV DNA positive [35].

For the anti-HBc non-reactive population, minipool testing was carried out by forming different sample pools. Each of the pool contained 1ml of plasma, which comprised of 100μl aliquots from 10 samples. Viral DNA was then isolated using the QIAamp UltraSens Virus kit (Qiagen, Hilden, Germany) followed by detection of HBV DNA in all pools. For subsequent identification of specific seronegative OBI samples, DNA from each of the 10 samples in a positive pool was individually re-extracted with QIAamp DNA Blood Kit (Qiagen, Hilden, Germany) using an initial plasma volume of 400μl and an elution volume of 80μl. It was then subsequently screened for HBV DNA *via* nested-PCR for two different HBV regions. This later strategy ensures that the detection limit for HBV DNA remains the same both in case of both anti-HBc positive and negative subjects. HBV DNA was quantified as per a method described earlier [36], and stringent precautions were taken to avoid cross contamination [37].

### PCR amplification, sequencing and cloning

The complete small surface (*S*) gene (nt 155–835 from *EcoRI* site), which also encompasses the partial overlapping polymerase gene (*P*), was amplified by hemi-nested PCR using primers HB1F (nt 18–39) and HS4R (nt 989–970) in the first round and B2 (nt 65–84) and HS4R (nt 989–970) in the second round (Amplicon length: 906 base pair) [34]. In addition, the complete HBV genome of 5 OBI subjects was successfully amplified and sequenced as reported previously [38]. All amplicons were sequenced bidirectionally using prism Big Dye kit (v3.0) and ABI 3130xl Genetic Analyzer (Applied Biosystems, Foster City, USA).

In order to elucidate the quasispecies variation among study subjects, 11 occult and 8 chronic HBV infected patients- all belonging to HBV/D—were selected. The *S* gene amplicons for the above isolates were cloned into the pGEM T Easy vector (Promega, Madison, Wisconsin) followed by sequencing of 10 clones per patient, as described for a previous study of OBI in the United States (US) [27].

All the accession numbers for the complete HBV *S* gene sequenced in this study— including clonal sequences—are as follows:

- OBI/HIV co-infection (HBsAg negative)—
  - KT366472 –KT366490; KT366512 –KT366613: Complete small *S* gene (including both dominant and clonal sequences).
  - KT366498 –KT366502: Whole genome of 5 OBI strains.
- HIV/HBV co-infection (HBsAg positive)—
  - KF798220—KF798258: Complete small *S* gene sequenced in our previous study [34].
  - KT366614 –KT366643: Complete small *S* gene newly sequenced in this study.
  - KT366644 –KT366720: *S* gene clonal sequences.

### Phylogenetic analysis and evaluation of genetic diversity

HBV genotypes/subgenotypes were evaluated based on the complete *S* gene using a Bayesian Markov chain Monte Carlo (MCMC) approach as implemented in the Bayesian Evolutionary Analysis by Sampling Trees (BEAST) v1.8.0 program [39] under an uncorrelated log-normal relaxed molecular clock and the Hasegawa, Kishino and Yano (HKY) model. A chain length of 50,000,000 with sampling every 5000^th^ generation was used. The effective sample size (ESS) was >500 for all the parameters, indicating sufficient sampling. Posterior probabilities >90% were considered statistically significant.

All study sequences were compared to non-recombinant references (A-H) to evaluate recombination using the Simplot software (v3.5.1) with a window size of 200 base pairs and a step size of 20 base pairs. The bootscan analysis was performed under the Neighbour-Joining method using 1000 bootstrap replicates and a parental threshold of 80%. In addition, the viral epidemiology signature pattern analysis (VESPA) (available at http://hiv-web.lanl.gov) tool was used to identify potential signature sequences associated with occult infection.

Intra- and inter-group evolutionary distance were calculated using Kimura two-parameter model [40], whereas the rates of non-synonymous (dN) and synonymous (dS) substitutions were calculated *via* the Nei–Gojobori method as implemented in the MEGA 5 software [41, 42]. Additionally, intra-patient Shannon entropy was calculated using the formula: S_n_ = -Ʃ(pi lnpi)/lnN, where pi is the frequency of each distinct nucleotide sequence, and N is the total number of sequences analyzed per patient.

### Mutational profiling

GenBank references for HBV subgenotype A1 (n = 15), D1 (n = 144), D2 (n = 36), and D3 (n = 30) were compared with the OBI surface gene sequences (clonal + dominant) in a subgenotype-matched manner to identify distinct substitutions in the *S* and reverse transcriptase (RT) regions. Mutations among the occult sequences with <10% frequency in the chronic HBV or reference sequences of respective subgenotype were considered to be associated with OBI.

### Statistical analysis

Determining the median of different parameters was done using Microsoft Excel. Two-tailed nonparametric test (Mann-Whitney) was performed for comparing the medians of continuous variables including age, CD4 T-cell count, ALT, and AST using Graphpad Prism program (version 4.0.3). Chi-square test was used to compare categorical data using StatCalc (EpiInfo version 6.0, Centers for Disease Control and Prevention and World Health Organization, Geneva, Switzerland). P-values ≤0.05 were considered statistically significant.

## Results

### Detection of occult HBV infection

**Fig 1:**
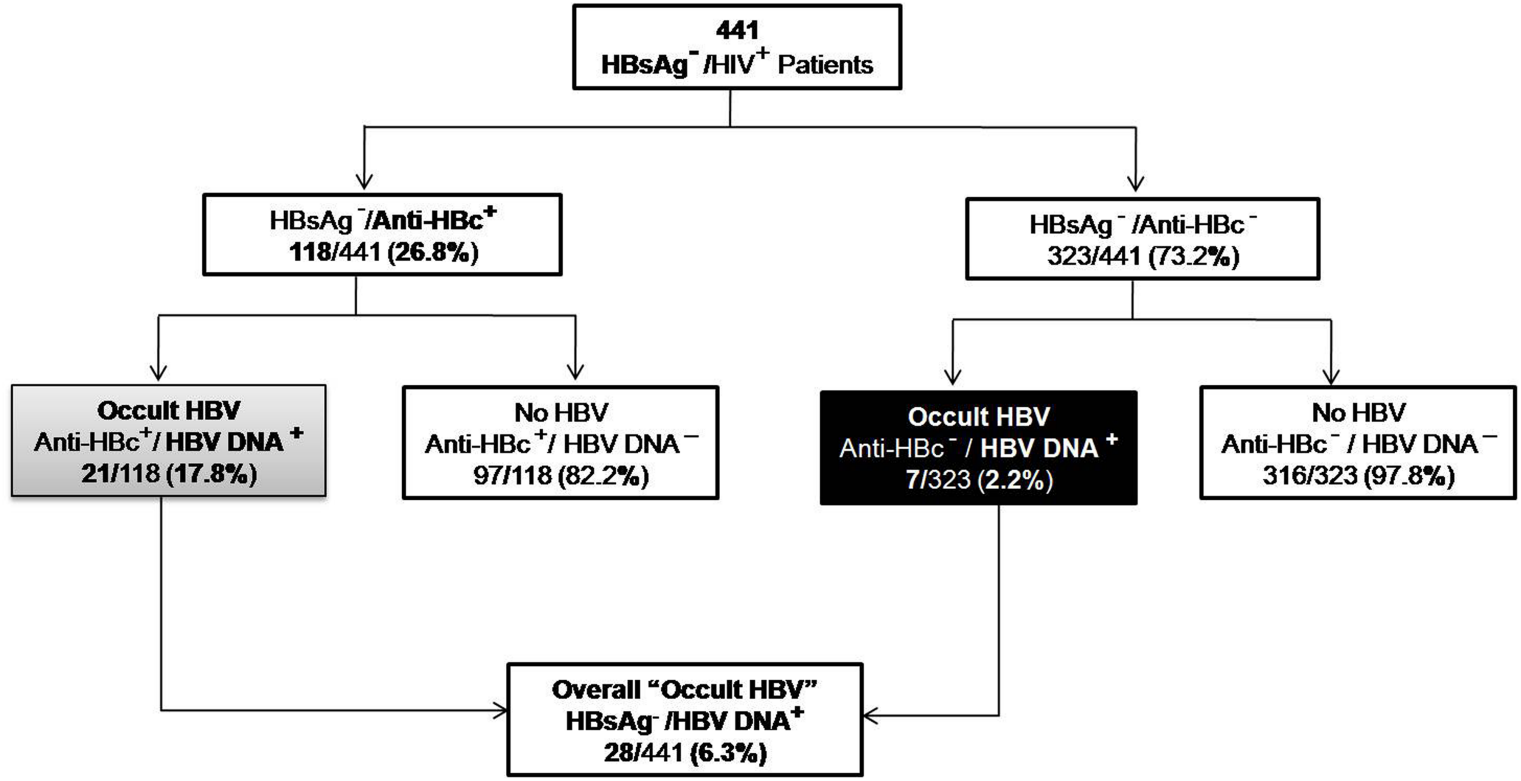
Identification of occult HBV infection among HIV treatment naïve patients. HBsAg negative/ HIV positive individuals were screened for the presence of HBV DNA to identify occult HBV infection. The HBsAg negative group was stratified into anti-HBc positive and anti-HBc negative groups.

In our previous study, 11.3% of the HIV infected patients from eastern India were chronically infected with HBV [30]. Hence, we evaluated the rate of OBI among the HBsAg negative/HIV-positive patients of the same cohort. 118 of the 441 HIV-infected individuals evaluated were anti-HBc positive (26.8%). 21 of 118 (17.8%) patients had detectable HBV DNA. Of the subjects with no evidence of any HBV serological markers, 7 of 323 (2.2%) were positive for HBV DNA. Thus, the overall rate of OBI was 6.3% (28 of 441) (Fig 1).

### Clinical characterization of the study population

These 28 individuals included 22 males and 6 females with the median age being significantly higher for males (39 years, inter-quartile range [IQR] 25–55 years vs. 32.5 years, IQR 22–41 years, p = 0.005). Moreover, 11 (39.3%) of the 28 occult samples contained both anti-HBs and anti-HBc, whereas 10 (35.7%) were anti-HBs negative but anti-HBc positive. Taken together, they constitute the “seropositive OBI” group (21/28, 75%), while HBV DNA positive samples lacking any serological marker(s) are termed “seronegative OBI”. Anti-HBs levels ranged between 17.75 mIU/ml and 1370 mIU/ml with a median of 112.75 mIU/ml. No statistically significant differences were observed between the seronegative and seropositive OBI groups with respect to CD4 T-cell count, ALT and AST levels.

The major baseline characteristics of the OBI/HIV+ and HIV mono-infected population are presented in Table 1. While not statistically significant, occult infection was associated with minor elevation in AST level (47.5 U/L, IQR 23–121 U/L vs. 41 U/L, IQR 14–344 U/L) and lower CD4 T-cell count (153 cells/mm^3^, IQR 6–510 cells/mm^3^ vs. 173 cells/mm^3^, IQR 2–1175 cells/mm^3^) compared to HIV mono-infected patients. Indeed, the majority of occult patients had CD4 T-cell count of <250 cells/mm^3^ (25/28, 89.3%) and were clinically asymptomatic with ALT levels of <50 U/L (19/28, 67.9%). Furthermore, the clinical parameters were also compared between the anti-HBc positives with and without detectable HBV DNA. Although no variables were significantly different, the median CD4 T-cell count was lower for patients who were positive for both anti-HBc and HBV DNA (142 cells/mm^3^, IQR 6–394 cells/mm^3^ vs. 158 cells/mm^3^, IQR 8–897 cells/mm^3^, p = 0.15).

**Table 1:** Baseline clinical and demographic characteristics.

Variable	Total	HIV positive (no occult or chronic HBV)	HIV positive/Occult HBV	p-value*
Patients, n	441	413	28	-
Age, years, median (IQR)	36 (16–70)	36 (16–70)	39 (22–60)	0.38
Male gender, n (%)	297 (67.3)	275 (66.6)	22 (78.5)	0.2
CD4 T-cell count, cells/mm^3^, median (IQR)	170 (2–1175)	173 (2–1175)	153 (6–510)	0.2
ALT, U/L, median (IQR)	30 (3–1647)	30 (3–1647)	35 (8–197)	0.5
AST, U/L, median (IQR)	41.5 (14–344)	41 (14–344)	47.5 (23–121)	0.06
HBV DNA, log_10_IU/ml, median (IQR)		-	1.7 (1.4–2.9)	-

*p-value for comparison between occult HBV/HIV^+^ and HIV mono-infected subjects; IQR, inter-quartile range

**Fig 2:**
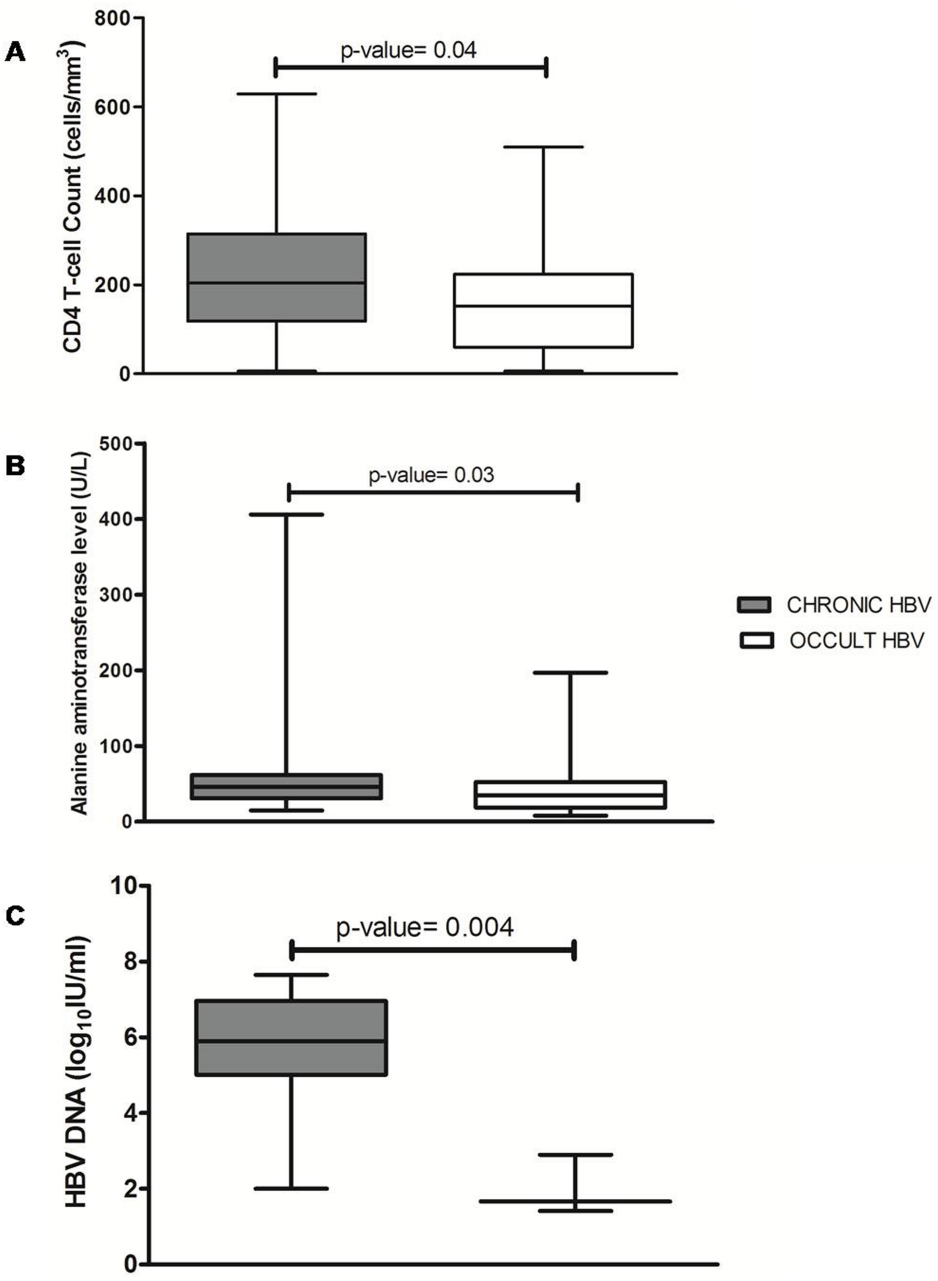
Comparative analysis of the clinical characteristics associated with chronic versus occult HBV infection in HIV-positive patients. (A) CD4 T-cell count, (B) ALT, and (C) HBV DNA titres are represented as box and whisker plots for both infection groups. The box represents the 25% to 75% inter-quartile (IQR) range, with a line at the median value, while the whiskers represent the lowest and the highest values. The CD4 status, ALT, and AST levels were significantly elevated among the chronic compared to the occult patients.

The demographic and clinical parameters of the HIV/HBV co-infected patients were also compared with the occult cases. There was a significant difference in HBV DNA titres between chronic and occult patients (5.9 logIU/ml, IQR 2–7.65 logIU/ml vs. 1.7 logIU/ml, IQR 1.4–2.9 logIU/ml, p = 0.004). Likewise, significantly higher levels of CD4 T-cell count (205 cells/mm^3^, IQR 6–629 cells/mm^3^ vs. 153 cells/mm^3^, IQR 6–510 cells/mm^3^, p = 0.037) and ALT levels (46 U/L, IQR 15–406 U/L vs. 35 U/L, IQR 9–197 U/L, p = 0.03) were observed among the HBsAg positive subjects compared to OBI (Fig 2).

### Phylogenetic analysis

**Fig 3:**
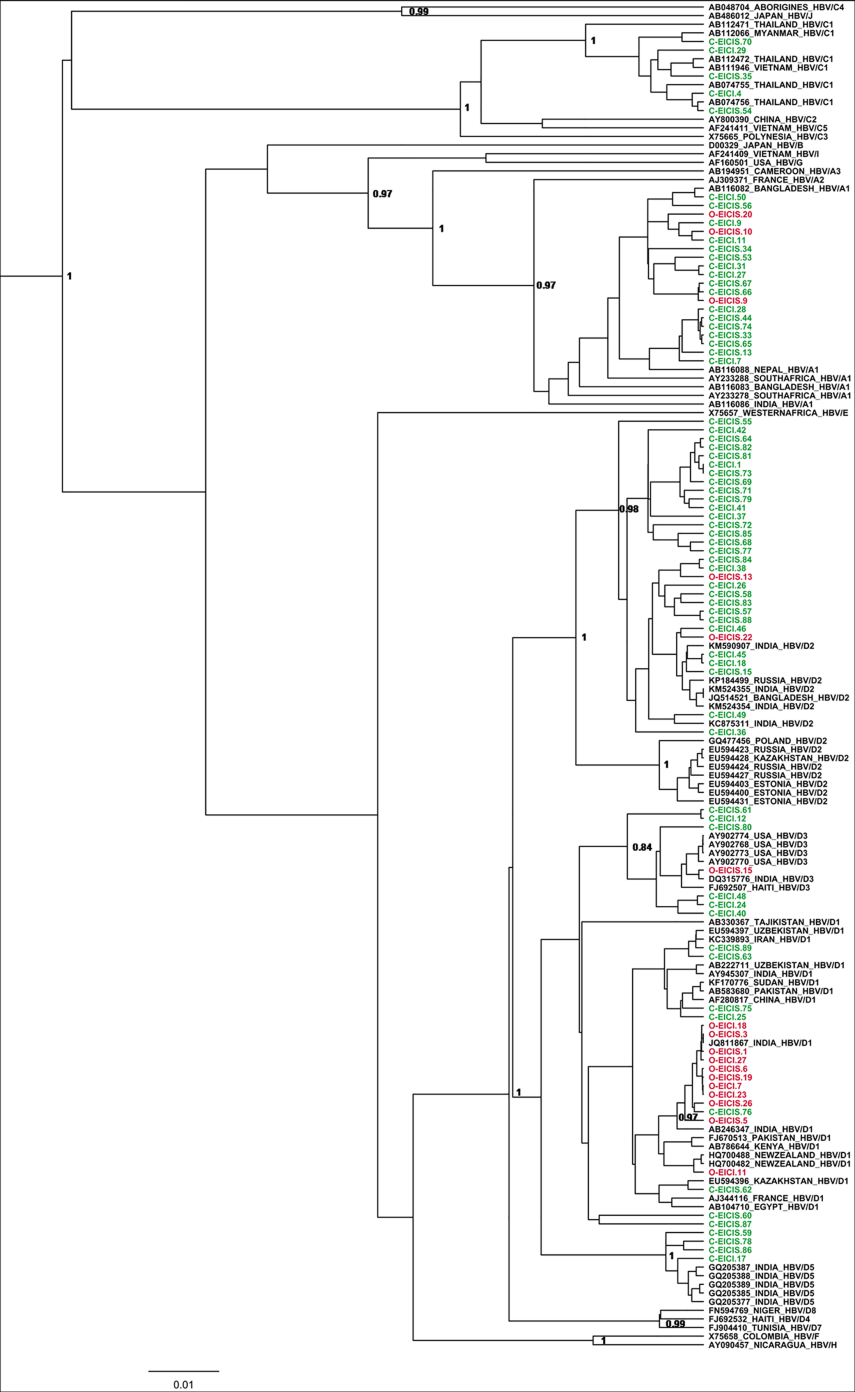
Phylogenetic analysis of occult and chronic HBV strains circulating among HIV-infected individuals in eastern India. The phylogenetic relatedness was analyzed by means of Bayesian Markovchain Monte Carlo (MCMC) approach using a chain length of 50,000,000 and sampling in every 5000^th^ generation and was based on the small surface (*S*) gene (nt 155–835 from *EcoRI* site). The occult HBV isolates (represented in red and labelled with **O-**) and the chronic HBV isolates (represented in green and labelled with **C-**) were analyzed with respect to GenBank reference sequences (represented in black) which are designated by their respective accession numbers along with their HBV genotypes/subgenotypes and country of origin. Posterior probabilities ≥0.9 are shown. HBV/D was the predominant genotype both among chronic (68.1%) and occult (82.4%) subjects, with HBV subgenotype D1 being widespread in OBI (64.7%) and D2 in chronic infection (42%).

69 representative HIV-positive individuals with chronic HBV and 17 with occult HBV were compared to 70 HBV reference sequences. HBV genotype D was the predominant genotype among the chronic (47/69, 68.1%) and occult subjects (14/17, 82.4%) (Fig 3). However, subgenotype D1 was most common in OBI (11/17, 64.7%), while D2 was more common during chronic infection (29/69, 42%). In fact, the frequency of HBV/D1 was significantly higher among the OBI population (64.7% vs. 11.6%; p = <0.0001), with few strains belonging to HBV/A1 (3/17, 17.6%) and none to HBV/C1.

**Fig 4:**
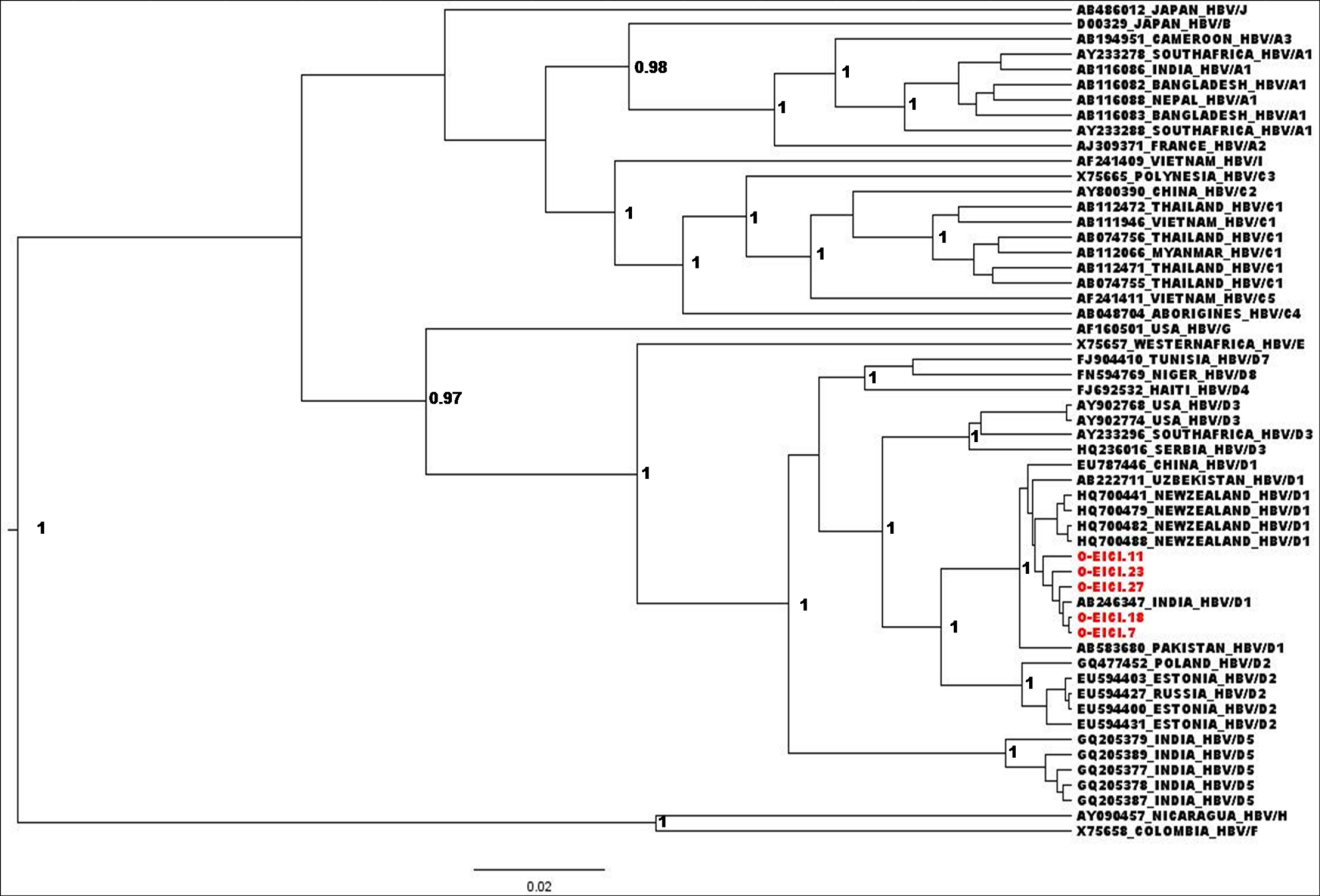
Relatedness of 5 occult HBV strains based on complete genome analysis. The phylogenetic analysis was performed by Bayesian inference using a chain length of 50,000,000 and sampling in every 5000^th^ generation. The occult HBV strains (represented by in red and also by **O**-) were compared to complete genome reference sequences from GenBank. Posterior probabilities ≥0.9 are shown.

The occult HBV/D1 isolates tended to cluster together with a mean intra-group nucleotide distance of 0.1% (Std Err = 0.05%) and were distinct from the chronic HBV/D1 sequences with the exception of C-EICIS.76 (Inter-group distance = 0.67%; Std Err = 0.22%). We further sequenced complete HBV genomes of 5 OBI subjects and a phylogenetic tree was constructed using 50 reference sequences (Fig 4). Similar results were obtained as all of them belonged to HBV/D1, with an overall mean nucleotide diversity of 0.6% (Std Err = 0.1%).

### Recombination and viral epidemiology signature pattern analysis (VESPA)

**Fig 5:**
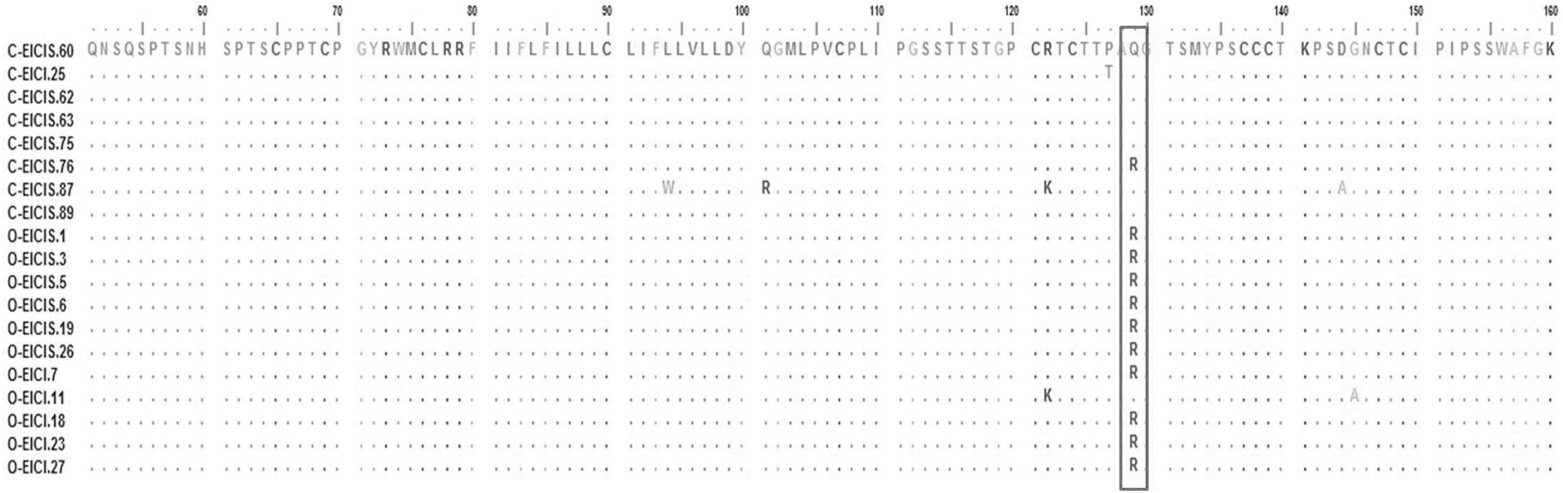
Signature pattern analysis of HBV surface gene between chronic and occult HBV/D1 strains found in HIV-positive individuals. The complete HBV small surface ORF was compared between chronic HBV/D1 (n = 8) and occult HBV/D1 strains (n = 11). The major substitution to be detected was at the codon 129 where a Glutamine to Arginine substitution took place. Occult HBV sequences are labelled as O-, whereas the chronic HBV sequences are labelled as C-. The analysis was performed with a threshold value of 0.9.

Recombinant analysis of the complete *S* gene was performed for all chronic, and the occult HBV sequences. No isolates showed any evidence of recombination. VESPA identified signature nucleotide substitutions including G226, G540, T619, and C820 (from *EcoR1* site) in the surface gene of occult compared to chronic HBV/D1 sequences. Of them, G540 was the only non-synonymous mutation that resulted in a Glutamine to Arginine change at the 129^th^ codon of *S* gene. 10 of 11 (91%) of the occult D1 sequences harbored this sQ129R mutation (Fig 5).

### Quasispecies diversity and immune selection pressure

**Fig 6:**
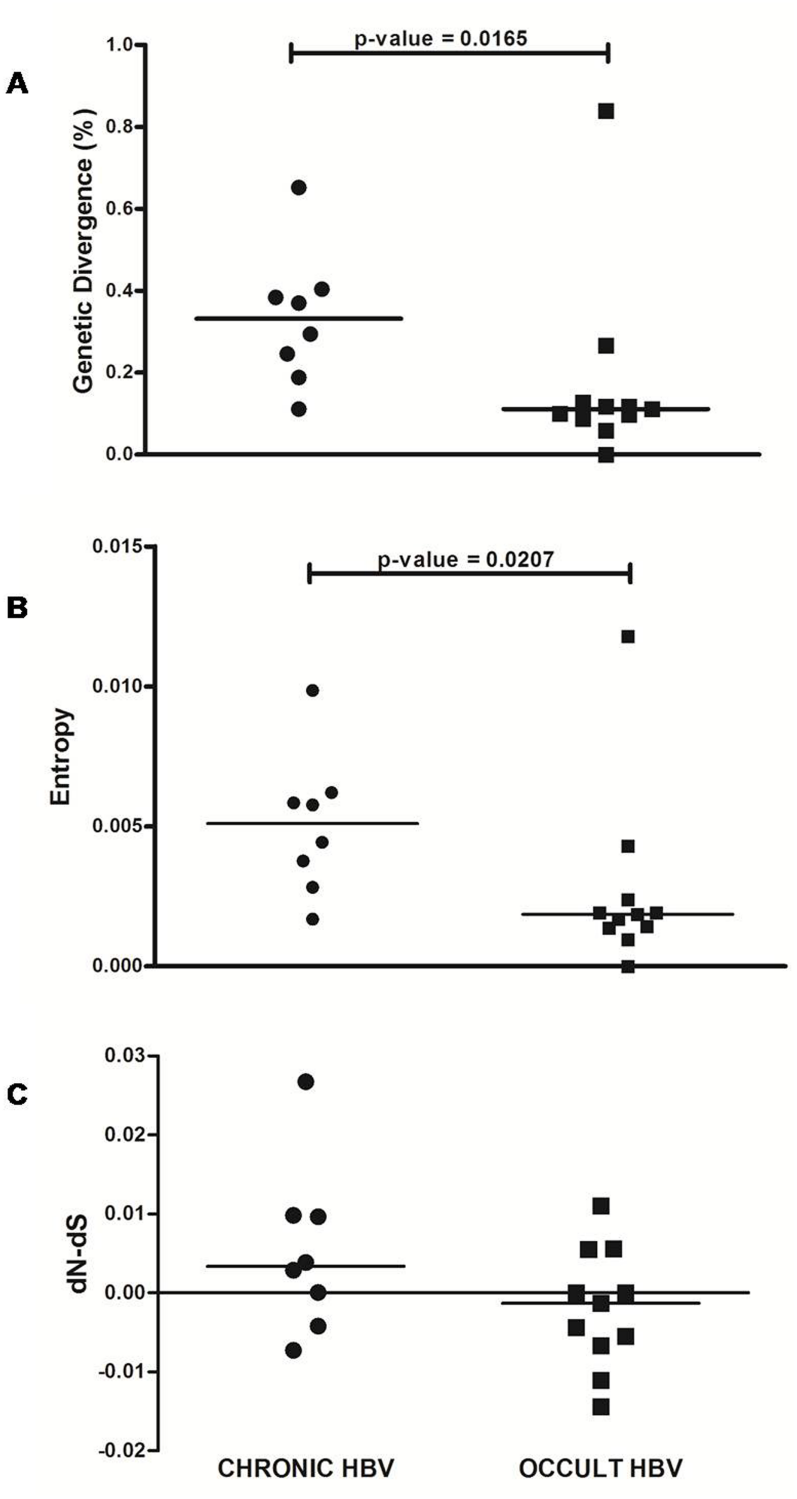
Intrapatient quasispecies diversity of the small surface ORF from chronic and occult HBV/D strains. Scatter Plots were generated for (A) genetic divergence (%), (B) Shannon entropy, and (C) dN-dS for both groups. 8 chronic and 11 occult HBV/D subjects were included with 10 clones from each individual was sequenced to evaluate quasispecies diversity. Each ■ and ● represents the mean of individual patient values for the occult HBV and chronic HBV groups, respectively, whereas the horizontal bars represent the overall median values for each group.

The intra-patient quasispecies analysis revealed mixed infection with distinct HBV subgenotypes (80% HBV/D1 and 20% HBV/D3) in one individual with OBI (O-EICI.23). Moreover, the chronic HBV/D subjects were associated with significantly higher median genetic diversity (0.33% vs. 0.11%; p = 0.0165) and median Shannon entropy (0.7 vs. 0.39; p = 0.0006) for the *S* gene compared to occult (Fig 6A and 6B). The median dN-dS value for the chronic HBV patients was 0.003 compared to -0.001 for OBI (Fig 6C), although this difference was not statistically significant.

### Mutational profiling of the occult HBV strains

68 occult-associated mutations were identified within the *S* and overlapping RT region (Table 2). The major mutation detected among occult D1 sequences was Q129R (58.8%) in the *S* gene and N33D (58.8%) in the RT region of the *P* gene. These mutations were found in all HBV/D1 sequences except O-EICI.11. Other diagnostic escape mutations found in the surface ORF were sR122K (5.9%), sG145A (5.9%), and sW172* (5.9%).

**Table 2:** Mutational analysis of the HBV polymerase and surface ORFs for the occult HBV subjects.

Sample ID	Subgenotype	Mutations in Reverse Transcriptase (RT)	Anti-HBV Drug Resistant Mutations	Mutations in Surface (S)	Vaccine/Diagnostic Escape Mutations
O-EICIS.1	D1	rtV27F, rtN33D*, rtY54L		sT46F, sQ129R*^#^	sQ129R
O-EICIS.3	D1	rtN33D*, rtW58G		sQ129R*^#^, sV177A^#^	sQ129R
O-EICIS.5	D1	rtN33D*, rtY54F, rtR110G*^#^		sT46S, sQ129R*^#^	sQ129R
O-EICIS.6	D1	rtN33D*, rtA200V*		sL98R, sQ129R*^#^, sL192F*^#^	sQ129R
O-EICI.7	D1	rtN33D*		sQ129R*^#^	sQ129R
O-EICI.11	D1	rtN53K^#^, rtY54L, rtM129T, rtA194V		sT45N, sT46S, sG112E^#^, sR122K*^#^, sG145A*^#^, sL186F	sR122K, sG145A
O-EICI.18	D1	rtN33D*, rtL140P, rtI162T, rtQ215R^#^		sL84P, sQ129R*^#^, sS132P, sS154P^#^, sV177A^#^, sS207G	sQ129R
O-EICIS.19	D1	rtN33D*, rtF46L, rtS105T		sL26P, sQ129R*^#^	sQ129R
O-EICI.23	D1 (8)	rtN33D*, rtL69P, rtL179P		sS61P^#^, sQ129R*^#^, sS171P	sQ129R
	D3 (2)	rtL91P^#^, rtH124N^#^, rtN131D		sF83L, sT125M^#^, sP127T^#^, sW172stop^#^	sW172stop
O-EICIS.26	D1	rtN33D*		sQ129R*^#^	
O-EICI.27	D1	rtK32E*, rtN33D*, rtF183C, rtA211T		sQ129R*^#^, sL175V	sQ129R
O-EICIS.13	D2	**rtN53D**^#^, **rtY54H, rtV103I**^#^		**sP135H**^#^, **sG185E**	
O-EICIS.22	D2	**rtN53D**^#^, **rtY54H, rtV103I**^#^, **rtE218A**		**sA128V**^#^, **sS210R**	
O-EICIS.15	D3	rtH124N*, rtN131D*, rtA186T, rtP196R		sL84P, sT125M*^#^, sP127T*^#^, sP188A	
O-EICIS.9	A1	**rtY126H**^#^, **rtL180M**^#^, **rtM204V**^#^	rtL180M, rtM204V	**sF134Y, sI195M**^#^	
O-EICIS.10	A1	**rtR110G**^#^, **rtY126H**^#^		**-**	
O-EICIS.20	A1	**rtY126Q**		**sQ101H**^#^, **sT118K**^#^, **sP142L**^#^	

Shown are the occult-associated mutations in the HBV reverse transcriptase and surface ORF. The *S* gene of the underlined patient IDs was cloned in order to evaluate their quasispecies diversity. All the dominant and clonal sequences were compared with the references of their respective subgenotypes. Mutations in bold indicate that their presence in the dominant viral strain.

* indicates that the mutations are present in more than one viral clone of the same patient.

^#^ indicates that the particular mutation has been reported previously.

Despite the patient population being ART-naive, one OBI sample (O-EICIS.9; belonging to HBV/A1) of 17 (5.9%) harbored the rtL180M+rtM204V lamivudine resistant mutations. In addition, on analyzing the complete genome of 5 occult HBV/D1 strains, it was found to be associated with mutations such as psA39T in *preS*, xP42T in *X*, and cT80I in the core gene. None of the occult sequences contained the basal core promoter (T1762/A1764) or precore (A1896) mutations.

## Discussion

Occult HBV infection poses a serious diagnostic challenge and has several clinical implications including secondary transmission, ALT elevations, and the development of HCC. However, the epidemiology and virologic characteristics of OBI are not well defined among the HIV infected population of India. The salient findings of this study include 1) high prevalence of occult infection among the anti-HBc positive subjects, as well as the presence of seronegative OBI, 2) distinct differences with respect to subgenotype distribution and clonal diversity between occult and chronic HBV infection, and 3) potential association of OBI with HBV/D1 harboring a signature sQ129R mutation in its surface gene.

Our previous report evidenced high rates of HIV/HBV co-infection (11.3%) in eastern India, which served as the basis for the current cross-sectional study on OBI. The overall frequency of occult infection in the HIV infected cohort was 6.3% with the rate being significantly higher among the seropositive (anti-HBc reactive) individuals compared to seronegative ones (17.8% vs. 2.2%; p-value = <0.0001) (Fig 1). This high rate of OBI among anti-HBc positive subjects was comparable to reports available from Sub-Saharan Africa [12, 13, 35]. In fact, other studies from India, even though scanty, had also reported a similar high rate of OBI among the HIV-positive population [31, 32, 43]. However, these were mostly prevalence-based studies, involving limited number of samples that did not explore the viral mutations associated with OBI in this cohort or only analyzed partial *S* gene sequences [33, 43]. Moreover, 39.3% of our occult samples carried detectable levels of anti-HBs, suggesting that the presence of anti-HBs is not always indicative of resolved infection. Rather in these patients, the presence of anti-HBs might facilitate the development of OBI by masking HBsAg and accelerating its elimination by macrophages [44].

Although seropositive OBI represents the majority occult cases, it should be highlighted that ~20% of occult infections are serologically negative as well [4]. In our current analysis, 7 OBI patients (2.2%) had no serological evidence of HBV infection. Similar reports are also available from South Africa (2.2%) and the US (0.55%) where a substantial proportion of the seronegative individuals were positive for HBV DNA [6, 35]. There is a possibility that these patients might be in the early window phase and as a result, lacks any HBV serologic markers. Nonetheless, no prior studies have reported the presence of seronegative OBI from India. Testing for HBV DNA in such patients is unlikely in our resource-limited infrastructure due to the asymptomatic nature of the patients and thus, results in an additional challenge for diagnosis and screening.

Generally, OBI is related to the intrahepatic persistence of the virus due to the strong suppression of viral replication and gene expression by the host defenses. It is occasionally or transiently detected in serum especially under conditions of immune-suppression but in very low amounts [22]. In this context, CD4 levels were lower for occult patients compared to both HIV mono-infection and HIV/HBV co-infection (Table 1 and Fig 2), with ~90% having a CD4 T-cell count of <250 cells/mm^3^. This is in accordance with previous reports on OBI demonstrating a similar association [6, 45, 46]. Further comparison between anti-HBc positives with and without detectable HBV DNA showed that the former group was associated with much lower CD4 count. It is plausible that in these OBI subjects, T-cells are not present in sufficiently high quantity to restrict HBV replication to the liver only; hence, HBV DNA was detectable in serum as well. Under such circumstances, there also remains a possible risk of reactivation of OBI into chronic HBV infection[22].

It is well established that HBV/D is the principal genotype in India and has a wide range of subgenotypes including HBV/D1, HBV/D2, HBV/D3, HBV/D4, HBV/D5, and HBV/D9 [47–49]. In our previous study, as well as in this analysis, HBV/D2 was the most common subgenotype among the HBsAg positive HIV-infected patients [34, 50]. The phylogenetic analysis of the occult HBV strains from HIV patients showed that ~82% of them belonged to HBV/D. However, they differed from their overt counterpart in the subgenotype distribution as majority of OBI/HIV+ isolates belonged to HBV/D1 (Fig 3). At the molecular level, the occult HBV/D1 strains were much more conserved and distinct from the chronic HBV/D1 strains isolated from HIV/HBV co-infected patients. Interestingly, previous studies from our lab reported high prevalence of HBV/D3 among OBI cases from blood donors without HIV infection [47, 51, 52]. These HBV/D3 isolates exclusively carried the sT125M mutation in their surface gene and were different from the HBV/D3 isolates found among HBsAg positive patients [47, 51]. Thus, the predominance of HBV/D1 among the OBI/HIV cohort of eastern India was rather unique. The possible reason for such drift in prevailing subgenotype remains unclear due to lack of sufficient data on OBI/HIV co-infection from India. It might be related to the influence of HIV on HBV epidemiology; however, this requires further investigation.

Relevant differences were also found regarding quasispecies variability with the HBsAg positive strains being associated with higher genetic distance and shannon entropy for the *S* gene as compared to OBI. In addition, the former subjects showed dN-dS value greater than zero suggesting that positive immune selection pressure is acting upon the surface gene (Fig 6). It has been shown that chronic HBV infection among HIV patients is associated with high levels of HBsAg, which might lead to increased immune responses [53, 54]. Hence, in order to evade this immune pressure, non-synonymous substitutions may occur within the surface gene resulting in such high dN-dS values, while the lack of HBsAg in OBI is responsible for their limited diversity. These results were consistent with another report from Ghana, which also demonstrated low quasispecies divergence among individuals with occult HBV genotype E infection [55]. In contrast, a US study of genotype A observed higher diversity in the OBI cases [27], suggesting the possibility of genotype-dependent differences in quasispecies diversity.

The possible mechanism(s) responsible for the lack of HBsAg in occult infection is debatable. It may be either attributed to reduced or no HBsAg expression, lowered secretion of HBsAg from hepatocytes and/or altered HBsAg antigenicity [26, 29, 56–58]. Common amongst these probable hypotheses is the presence of several mutations within the HBV genome, particularly with the envelope gene. In line with this, we identified 68 different mutations encompassing the *S* and its overlapping *P* gene (Table 2). Of the many mutations that were detected, the major substitution to be associated with occult infection was sQ129R in the surface gene (Table 2). This mutation was exclusively found in HBV/D1 isolates, with ~90% of the occult D1 strains carrying it (Fig 5). It had been previously reported to be associated with diagnostic escape and perinatal transmission [59]. In one instance, *Blaich et*.*al* reported reactivation of HBV infection with mutated HBsAg harboring sQ129R in a liver transplant recipient, who previously received a graft from a complete seronegative individual [60]. In fact, an *in vitro* study showed that this mutation significantly impairs the secretion of surface protein from hepatocytes [29], thus providing a probable rationale behind the lack of HBsAg in the OBI population.

Further sequence analysis revealed other mutations such as sR122K, sG145A andsW172* within the surface ORF of the OBI subjects (mainly belonging to HBV/D1 and D3) but at low frequencies. The presence of both sR122K and sG145A had been earlier reported among the occult patients from the US, where the combined presence of these two mutations resulted in decreased levels of HBsAg *in vitro* [26]. On the other hand, some HBV/A1 isolates carried the sT118K mutation in their major hydrophilic loop, which has been previously reported to be associated with failure in HBsAg detection [8]. Notably, two of the occult subjects belonged to HBV/D2, which was mainly predominant among HBsAg positive HIV cases [34]. The possible reason behind the lack of HBsAg in HBV/D2 isolates is not explicit. The presence of sA128V substitution, an immune escape mutation [61], is often associated with HBV/D2 and is considered as a subgenotype specific change [62]. It is unlikely that this mutation would render HBsAg undetectable as majority of the chronic D2 cases of this present study exhibited HBsAg positivity. Therefore, there is a possibility that the host defense may play a pivotal role in such cases.

Furthermore, 3TC resistance mutations (rtL180M + rtM204V) were also observed in one of the OBI patients, despite the population being treatment-naive. Even though such findings are rare, a similar report is also available from South Africa where ~30% of the naive occult HBV/HIV co-infected patients harbored 3TC resistance [63]. Previously, we observed high prevalence of 3TC resistance among the HIV/HBV co-infected subjects of eastern India who are on prolonged ART [64]. Therefore, the presence of such drug resistant strains in naive patients suggests that a probable transmission might have taken place from this high-risk cohort and it calls for immediate medical attention. Additional mutations were also identified within the *preS*, *X* and core gene of the OBI isolates. However, the clinical significance of these mutations is unknown.

In conclusion, our current study is one of the first from India to highlight the different facets of OBI in HIV infected cohort, where it acts as a potential threat in disguise. Even though screening of OBI is rare in our resource-limited settings, the results are of grave concern as high rate of occult infection was identified among the HIV infected population. The pattern of occult infection was found to be distinct from that of overt, especially with respect to subgenotype distribution or quasispecies diversity. The occult subjects were mainly of HBV/D1 and harbored a unique sQ129R mutation in their surface gene, which might affect their HBsAg secretion [29]. Hence, the current study helps us to decipher the challenges presented by OBI and stresses on the essentiality of its screening in the HIV cohort. This would further help to improve the overall management of the OBI/HIV co-infected patients either through regular follow-ups or if required, *via* antiviral treatment.

## Abbreviations

anti-HBc: antibody to hepatitis B core antigen
anti-HBs: antibody to hepatitis B surface antigen
ART: Anti-retroviral therapy
HBsAg: Hepatitis B surface antigen
HBV: Hepatitis B virus
HIV: Human immune-deficiency virus
OBI: Occult HBV infection
ORF: Open reading frame
